# Higher frequency of regulatory T cells in granulocyte colony-stimulating factor (G-CSF)-primed bone marrow grafts compared with G-CSF-primed peripheral blood grafts

**DOI:** 10.1186/s12967-015-0507-z

**Published:** 2015-05-07

**Authors:** Xiang-Yu Zhao, Yu-Tong Wang, Xiao-Dong Mo, Xiao-Su Zhao, Ya-Zhe Wang, Ying-Jun Chang, Xiao-Jun Huang

**Affiliations:** Peking University People’s Hospital and Peking University Institute of Hematology, Beijing Key Laboratory of Hematopoietic Stem Cell Transplantation, No. 11 Xizhimen South Street, Beijing, 100044 China; Peking-Tsinghua Center for Life Sciences, Beijing, 100871 China

**Keywords:** Regulatory T cells, Effector T cells, G-BM, G-PB

## Abstract

**Background:**

Regulatory T cells (Treg) in allografts are important for the prevention of graft-versus-host disease (GVHD) post-transplantation. The aim of this study was to compare the contents of Tregs and effector T cells in granulocyte colony-stimulating factor (G-CSF)-primed bone marrow grafts (G-BM) and peripheral blood grafts (G-PB).

**Method:**

G-BM and G-PB were obtained from 20 allogeneic donors. T-cell subgroups, including conventional T cells and different types of Treg cells, as well as the percentage of Ki67 expression on CD4^+^CD25^high^Foxp3^+^ Treg cells, were analyzed using flow cytometry. The levels of interferon-γ (IFN-γ) and interleukin-17 (IL-17) secreted by T cells stimulated with PMA and ionomycin were also determined by flow cytometry.

**Results:**

The percentage of CD4^+^CD25^high^CD127^-/dim^CD62L^+^ Treg cells was significantly higher in the G-BM group, with higher proportions of CD45RA^+^ naïve Treg cells and higher expression of CD69 on Treg cells in G-BM (*P* < 0.05). The percentage of Ki67 expression in CD4^+^CD25^high^Foxp3^+^ Treg cells in G-BM was significantly higher than that on G-PB. The suppressive functions of Treg cells in inhibiting T-cell activation were comparable between G-BM and G-PB. The proportions of CD4^+^CD25^−^CD69^+^ Treg subsets as well as Th1 cells in G-BM were also significantly higher than those in G-PB (*P* < 0.001). The proportions of conventional T cells and Th17 effector cells were comparable in G-BM compared with those in G-PB. Thus, the ratio of conventional T cells and CD4^+^CD25^high^CD127^-/dim^ regulatory T cells were lower in G-BM than that in G-PB (*P* = 0.014).

**Conclusion:**

In addition to the much higher T-cell counts in G-PB grafts that may contribute to more severe GVHD, the higher frequency of Treg cells and lower ratio of conventional T cells to Treg cells in G-BM compared with G-PB grafts might reduce GVHD post-transplantation in G-BM compared with G-PB transplantation.

**Electronic supplementary material:**

The online version of this article (doi:10.1186/s12967-015-0507-z) contains supplementary material, which is available to authorized users.

## Introduction

Allogeneic hematopoietic stem cell transplantation (allo-HSCT) is the only curative method available for malignant hematologic diseases. However, its broad application is limited by the high incidence of graft-versus-host disease (GVHD). The current allo-HSCT procedures consist mostly of bone marrow (BM) cells or granulocyte-colony stimulating factor (G-CSF)-primed peripheral blood stem cells (G-PB) or G-CSF-primed bone marrow (G-BM). Although both G-BM and G-PB contain large numbers of mature donor T cells that could cause GVHD [1], clinical data have demonstrated that patients undergoing G-PB transplantation were more likely to acquire severe acute GVHD refractory to prednisone and chronic GVHD (cGVHD) with a prolonged requirement for immunosuppression therapy to control symptoms compared to G-BM transplantation [2]. However, the underlying mechanism remains undefined.

CD4^+^CD25^high^CD127^-/low^ regulatory T cells (Treg cells) have been demonstrated to have immunosuppressive ability and to be key players in the regulation of immune responses [3]. Rezvanietal et al. determined that increased frequencies of CD4^+^Foxp3^+^ Treg cells in the peripheral blood of the donor negatively correlated with the incidence of GVHD in the graft recipient [4]. Subsequent studies have confirmed this correlation in the recipients of HLA-identical sibling and unrelated donor stem cell grafts [5,6], indicating that infused donor Treg cells in graft contents appear to lessen the severity of GVHD. In addition, donor immunoregulatory T cells, including CD4^+^CD25^high^CD62L^+^ regulatory T cells and CD4^+^CD25^−^CD69^+^ T cells, also contribute to decreased acute GVHD post-transplantation [7-9]. The CD4^+^CD25^high^CD62L^+^ T regulatory cell subset has optimal suppressive and proliferative potential. The CD62L^+^ cell subset is a more potent suppressor than the CD62L^−^ population or unfractionated CD4^+^CD25^+^ Treg cells [10]; therefore, only the CD62L^+^ subpopulation of CD4^+^CD25^+^ regulatory T cells protects from lethal acute GVHD [11]. Furthermore, CD69 is generally regarded as an activating marker, but recent studies have shown that CD69 is an immunoregulatory molecule induced following activation [12]. An additional report demonstrated that CD4^+^CD25^−^CD69^+^ T cells act as a new subset of regulatory CD4^+^ T cells characterized by a lack of Foxp3 expression and IL-10 secretion but with a high expression of CD122 and membrane-bound TGF-beta1 [13].

Previous work has demonstrated that the in vivo application of G-CSF would decrease the number of Treg cells in the bone marrow and increase the number of Treg cells in the peripheral blood [14-17]. In addition, our previous reports have demonstrated that differences existed in the immunological status between G-BM and G-PB [14,15,18,19]. However, until now, the Treg contents and function of G-BM and G-PB have not been comparatively analyzed. The aim of this study was to explore the contents of regulatory T cells, as well as the balance between the effector and regulatory arms of the immune system, including conventional T cells, Th17, and Th1 cells, between G-BM and G-PB.

## Methods

### G-CSF treatment of healthy donors and sample collection

G-BM and G-PB were obtained from 20 consecutive allogeneic donors. As shown in Table 1, this group of donors, 11 men and 9 women, provided informed consent and had a median age of 29 years ranging from 18 to 54 years. Approval for this study was obtained from the Institutional Review Board and Ethical Committee of the Health Center at Peking University. Donors received recombinant G-CSF (filgrastim; Kirin Co., Ltd., Tokyo, Japan) at a dosage of 5 μg/kg/d for 5 consecutive days. G-BM was collected on the 4th day of treatment by aspiration, and G-PB was obtained on the 5th day by leukapheresis using a continuous-flow blood cell separator (Gambro BCT, Lakewood, CO, USA; or Baxter, Chicago, IL, USA). The reason for using this protocol was that patients in our institute receive transplants composed of G-BM plus G-PB, which are harvested on days 4 and 5, respectively [20,21].

**Table 1:** **Donor characteristics**

**Characteristics**	**Total (n = 20)**
Median age at transplantation, years old (range)	29 (18–54)
Male sex, no. (%)	11 (55%)
Percentage of each T cell subset in CD4+ T cells before G-CSF in the peripheral blood (mean ± SE)	
CD4^+^CD25^high^CD127^-/low^ regulatory T cells	3.88 ± 0.48
CD4^+^CD25^high^CD62L^+^ regulatory T cells	3.05 ± 0.52
CD4^+^CD25^−^CD69^+^ regulatory T cells	0.15 ± 0.05
CD4^+^CD25^high^CD127^+^ conventional T cells	71.93 ± 1.90
Th1 cells	39.84 ± 5.02
Th17 cells	3.14 ± 0.39

### Immunophenotyping, intracellular staining and multiparameter flow cytometric analysis

Samples were stained without further separation to minimize selective losses shortly after collection. Combinations of the directly conjugated monoclonal antibodies CD3-V500, CD4-PerCP, CD56-PE, CD127-BV421, CD25-PE-CY7, CD25-APC, CD62L-FITC, CD69-APC, CD45RA-FITC, CCR7-PE, Ki67-FITC, Foxp3-APC (BD Bioscience, Mountain View, CA, USA), CD16-APC/CY7, HLADR-APC/CY7 (Biolegend, San Diego, CA, USA) and their relative allotypes were used in individual 8-color flow cytometry assays to analyze the immunophenotype of regulatory T cells and effector T cells.

Intracellular staining was performed using the Intracellular Staining Kit (eBioscience, San Diego, CA, USA). The expression of Ki67 was determined in freshly isolated CD4^+^CD25^high^Foxp3^+^ regulatory T cells. The cellular secretion and function of cytokines were determined after incubation of cells for 5 h with phorbol myristate acetate (PMA) (100 ng/ml) plus ionomycin (2 ug/ml, all reagents from Sigma Chemical Co., St. Louis, MO, USA) to stimulate maximal production of IL-17 and IFN-γ; GolgiStop (0.7 μl/ml) was added to the samples during the last 4 hours to sequester the proteins in the cytoplasm [22,23].

### Treg cell ex vivo suppression assay

We isolated human Treg (CD4^+^CD25^+^) and Tresp cells (CD4^+^CD25^−^) from G-BM and G-PB using a human CD4^+^CD25^+^ Regulatory T cell Isolation kit (Miltenyi Biotec). Tresp cells were labeled with 5 μM 5,6-carboxyfluorescein diacetate succinimidyl ester (CFSE) (Biolegend). A total of 1 × 10^4^ CFSE-labeled Tresp cells were cultured with unlabeled autologous Treg cells at different ratios, including 1:1, 2:1, and 4:1, in round-bottom 96-well plates in the presence of Treg Suppression Inspector consisting of Anti-Biotin MACSiBead Particles that were pre-loaded with biotinylated CD2, CD3, and CD28 antibodies (Miltenyi Biotec). After 5 days, an analysis of cell division was performed using the FACSCanto II system (BD Biosciences). The suppressive capacity of Treg cells toward the cocultured responder cells is expressed as the relative inhibition of the percentage of CFSE low cells [100 × (1–% CFSE low Tresp cells in coculture/% CFSE low Tresp alone)].

### Statistical analysis

To test the differences in the regulatory T cells and effector T cells between donors treated with G-BM and G-PB, a Wilcoxon signed-rank test or paired-sample T test was used. P < 0.05 was considered statistically significant.

## Results

### Comparison of multiple regulatory T cells between G-BM and G-PB

**Figure 1:**
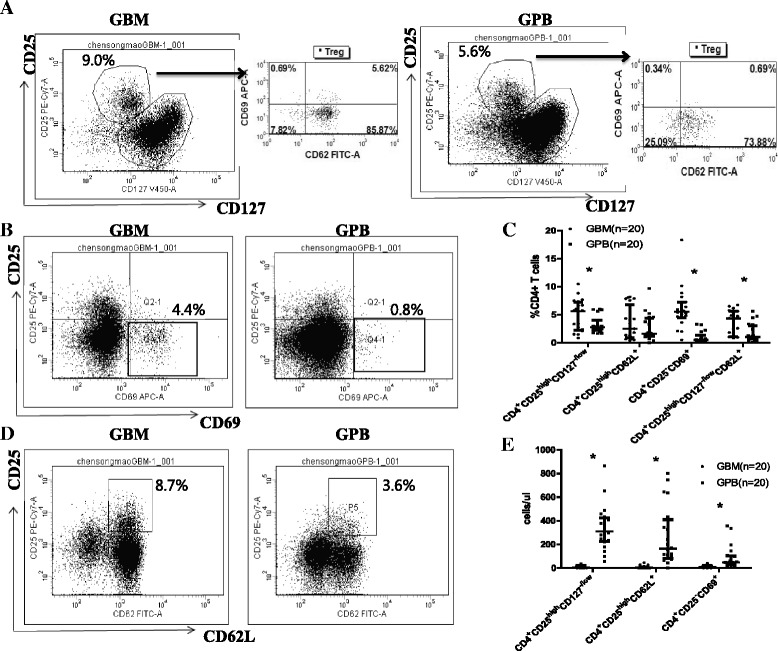
Comparison of regulatory T cells between G-BM and G-PB. Panel **A** shows representative plots of CD4^+^CD25^high^CD127^-/low^ regulatory T cells as well as CD69 and CD62L expression on CD4^+^CD25^high^CD127^-/low^ regulatory T cells. Panels **B** and **D** show the flow cytometry data for the expression of CD4^+^CD25^high^CD62L^+^ regulatory T cells as well as CD4^+^CD25^−^CD69^+^ regulatory T cells in G-BM and G-PB gated on CD4^+^ T cells. Panels **C** and **E** show a comparison of the percentage (%CD4^+^ T cells) and the absolute numbers (cells/μl) of the three types of regulatory T cells mentioned above as well as CD4^+^CD25^high^CD127^-/low^CD62L^+^ in G-BM and G-PB gated on CD4^+^ T cells (n = 20). *P < 0.05 by Wilcoxon signed-rank test (median with interquartile range).

The cartoon in Additional file 1: Figure S1 addresses the multiple alternative definitions of regulatory T cells and provides a heuristic overview of how these various subsets of T cells are related in ontogeny or in functional capacities. The percentage of CD4^+^CD25^high^CD127^-/dim^ regulatory T cells and CD4^+^CD25^−^CD69^+^ regulatory T cells among CD4^+^ T cells were significantly higher in G-BM compared to G-PB (4.97% ± 2.72% vs. 3.16% ± 1.52%, n = 20, p = 0.001; 6.27% ± 3.94% vs. 0.89% ± 0.92%, p < 0.001, Figure 1A, B and C). No statistically significant differences were found between G-BM and G-PB in the proportions of CD4^+^CD25^high^CD62L^+^ regulatory T cells among CD4^+^ T cells (2.54% ± 2.23% vs. 3.41% ± 2.89%, p = 0.204, Figure 1C and D). However, because of the significantly higher numbers of CD4^+^ T cells in G-PB compared to G-BM, the absolute numbers of CD4^+^CD25^high^CD127^-/low^ regulatory T cells, CD4^+^CD25^high^CD62L^+^ regulatory T cells and CD4^+^CD25^−^CD69^+^ regulatory T cells were significantly lower in G-BM than in G-PB (P < 0.0001, Figure 1E).

Considering that CD62L (L-selectin) is an important T-cell homing receptor as well as a marker for T-cell development and that CD69 is an important activating receptor that exerts regulatory functions in the immune response of T cells, we also compared differences in the expression of CD62L and CD69 on CD4^+^CD25^high^CD127^-/low^ regulatory T cells between G-BM and G-PB. Although the expression of CD62L was comparable in CD4^+^CD25^high^CD127^-/low^ regulatory T cells (59.88% ± 25.07% vs. 54.96% ± 20.71%, Figure 1A), the expression of CD69 was significantly higher in CD4^+^CD25^high^CD127^-/low^ regulatory T cells in G-BM compared with G-PB (2.38% ± 2.21% vs. 0.78% ± 0.50%, p = 0.005, Figure 1A). In addition, most of the CD4^+^CD25^high^CD62L^+^ regulatory T cells were CD127 negative. The percentage of CD127 negative cells among CD4^+^CD25^high^CD62L^+^ regulatory T cells was comparable between G-BM and G-PB (83.74% ± 13.69% vs. 85.33% ± 9.71%, p = 0.936). Therefore, the percentage of CD4^+^CD25^high^CD127^-/dim^CD62L^+^ regulatory T cells was significantly higher in G-BM compared to G-PB (3.43% ± 2.18% vs. 1.69% ± 1.54%, p = 0.006, Figure 1C).

**Figure 2:**
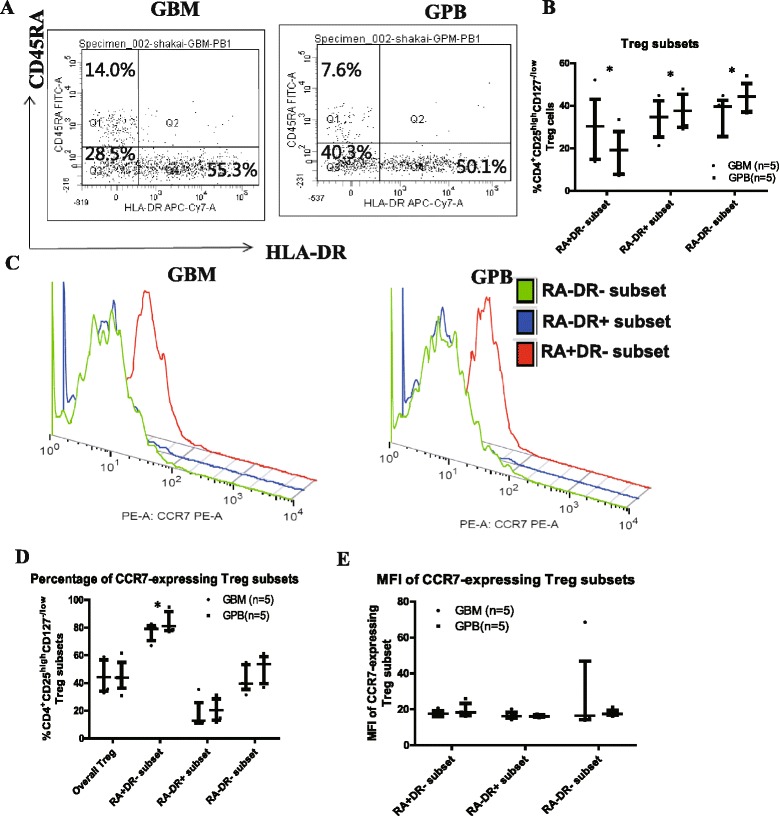
Comparison of regulatory T cell subsets between G-BM and G-PB. Panels **A** and **B** show representative plots and a comparison of 3 distinct Treg populations (CD45RA^+^HLADR^−^, CD45RA^−^HLADR^+^, CD45RA^−^HLADR^−^) in G-BM and G-PB gated on CD4^+^CD25^high^CD127^-/low^ regulatory T cells (n = 5); Panels **C**, **D** and **E** show representative plots and a comparison of the differences in the percentage of CCR7 expression and MFI among 3 Treg populations (n = 5). *P < 0.05 by Wilcoxon signed-rank test (median with interquartile range).

A phenotypic analysis of the regulatory T cell compartment revealed 3 distinct Treg populations (CD45RA^+^HLADR^−^, CD45RA^−^HLADR^+^, CD45RA^−^HLADR^−^) in G-BM and G-PB [24]. The percentage of CD45RA^+^HLADR^−^ naive Treg cells among the Treg cells was significantly higher in G-BM compared to G-PB (29.26% ± 15.50% vs. 18.06% ± 10.85%, P = 0.009, Figure 2A-B). However, the percentage of the CD45RA^−^HLADR^+^ Treg subset (34.01% ± 9.17% vs. 37.61% ± 7.99%, P = 0.028) and the CD45RA^−^HLADR^−^Treg subset (35.15% ± 8.98% vs. 43.83% ± 7.20%, P = 0.004) was significantly lower in G-BM compared to G-PB (Figure 2A-B). We also observed that the expression of CCR7 in CD45RA^+^HLADR^−^ Treg cells was significantly higher than that in CD45RA^−^HLADR^+^ Treg or in CD45RA^−^HLADR^−^ Treg cells (Figure 2C-D). The expression of CCR7 in Treg cells overall was comparable in G-BM and G-PB. However, the percentage of CCR7-expressing CD45RA^+^HLADR^−^ naive Treg cells in G-BM was significantly lower compared to those in G-PB (76.64% ± 6.23% vs. 84.02% ± 7.43%, P = 0.025, Figure 2C-D), but the MFI of CCR7-expressing CD45RA^+^HLADR^−^ naive Treg cells was comparable (Figure 2E).

**Figure 3:**
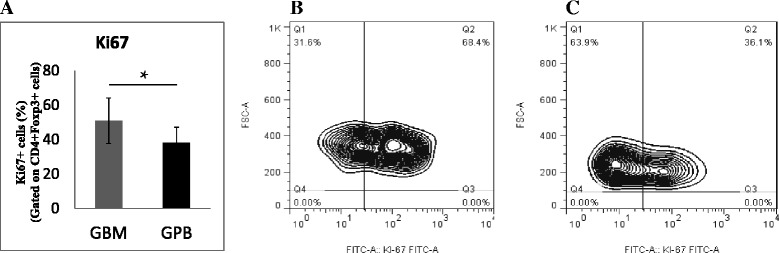
Comparison of the proliferation of regulatory T cell subsets between G-BM and G-PB. Panel **A**. Percentage of Ki67 expression on freshly separated CD4^+^CD25^high^Foxp3^+^ Treg cells in G-BM and G-PB (n = 12). Panels **B** and **C**. Flow cytometry plots (the percentage of Ki67 Treg cells is indicated above the boxes) from the representative G-BM **(B)** and G-PB **(C)**. *P ≤ 0.05 by Wilcoxon signed-rank test (mean ± SD). FSC, forward scatter.

The expression of Ki67 in CD4^+^CD25^high^Foxp3^+^ Treg cells was measured as a marker of cell proliferation. The results from the entire cohort of patients and a representative patient showed that CD4^+^CD25^high^Foxp3^+^ Treg cells in G-BM had higher levels of proliferation than those in G-PB (50.93% ± 13.25% vs. 38.19% ± 8.95%, P = 0.037, Figure 3).

### Comparison of the in vitro suppression function of regulatory T cells between G-BM and G-PB

**Figure 4:**
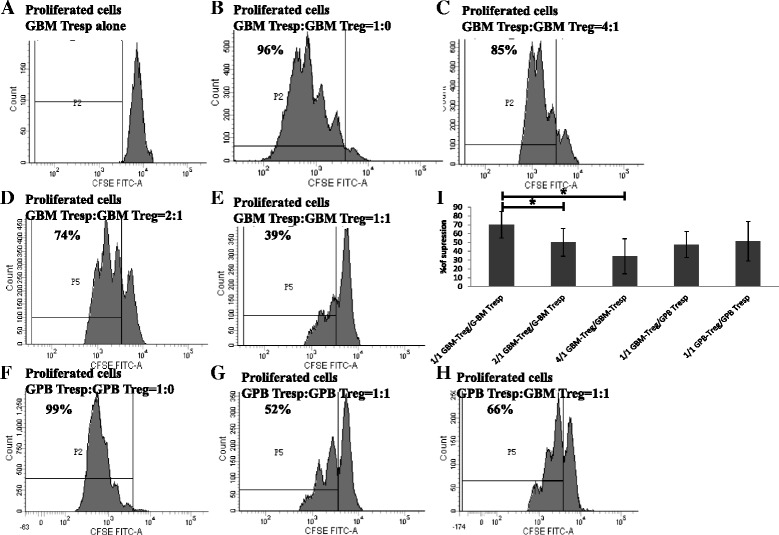
Comparison of the in vitro suppression function of regulatory T cell subsets between G-BM and G-PB. Panels **A-D**. Representative flow cytometry plots of the proliferation of CFSE-labeled responder T cells in G-BM without **(A)** or with Treg Suppression Inspector (Miltenyi Biotec) co-cultured without **(B)** or with an increasing number of Treg cells at ratios of 4:1 **(C)**, 2:1**(D)**, and 1:1**(E)** of Tregs in G-BM. Panels **F-H**. Proliferation of CFSE-labeled responder T cells in G-PB with Treg Suppression Inspector (Miltenyi Biotec) co-cultured without **(F)** or with equal numbers of Treg cells from G-PB **(G)** or G-BM **(H)**. Panel **I**. Suppression functions of Treg cells in inhibiting T-cell activation. G-BM-Treg indicates Tregs from G-BM, G-BM Tresp indicates Tresp from G-BM, G-PB-Treg indicates Tregs from G-PB, and G-PB Tresp indicates Tresp from G-PB (n = 5). *P < 0.05 by Wilcoxon signed-rank test (mean ± SD).

To compare the suppressive activity of Treg cells between G-BM and G-PB, we performed additional functional studies. The suppression assay was performed with a dilution series ranging from a ratio of 1:1 to 4:1 of G-BM-Tresp cells:G-BM-Treg cells or of G-PB-Tresp cells:G-PB-Treg cells as outlined in Figure 4. As an additional control, G-BM- or G-PB-Tresp were cultured alone with and without the Treg Suppression Inspector. In addition, G-BM-Treg cells were also cocultured with equal numbers of G-PB-Tresp cells in comparison to G-PB-Treg cells cocultured with equal numbers of G-PB-Tresp cells. As shown for a representative patient in Figure 4A-H, Treg cells suppressed the proliferation of CFSE-labeled responder T cells from the same donor, achieving the maximum suppression at a 1:1 ratio of Tresp to Treg cells. At a 1:1 ratio of Tresp to Treg cells, no statistically significant differences were found in the suppression capability of Treg cells among G-BM-Treg cocultured with G-BM-Tresp (70.13% ± 15.12%), G-BM-Treg cocultured with G-PB-Tresp (47.63% ± 14.73%), or G-PB-Treg cocultured with G-PB-Tresp (51.36% ± 22.29%) (Figure 4I). Significant differences in the suppression rates were found at a G-BM Tresp:G-BM Treg ratio between 4:1 and 1:1 or 2:1 and 1:1 (P = 0.043 and P = 0.043, respectively). Finally, a trend towards a higher suppression rate at a G-BM Tresp:G-BM Treg ratio of 2:1 compared to 4:1 (P = 0.080) was observed (Figure 4I).

### Comparison of the ratio of regulatory T cells and effector T cells between G-BM and G-PB

**Figure 5:**
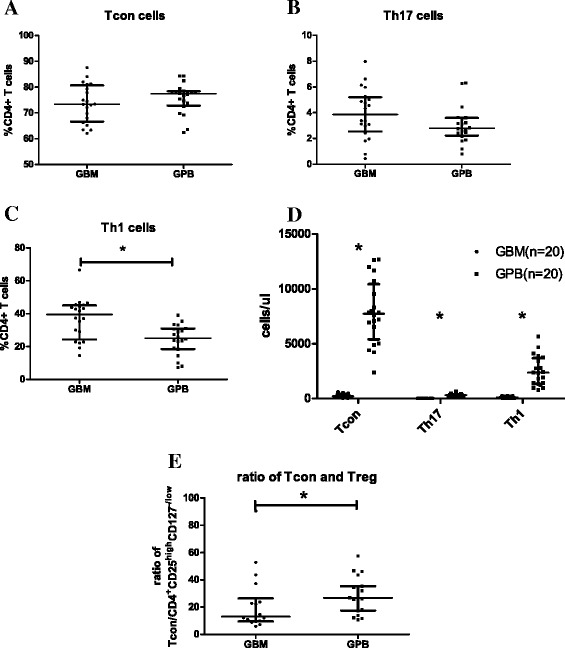
Comparison of effector T cells between G-BM and G-PB. Panels **A**-**C** show the percentage of CD25^low/-^CD127^+^ expression (**A**, Conventional T cells, Tcon), IL17 secretion (**B**, Th17 cells), and IFN-γ secretion (**C**, Th1 cells) on freshly separated CD4^+^ cells in G-BM and G-PB (n = 20). Panel **D** shows the comparison of the absolute numbers (cells/μl) of Tcon, Th17, and Th1 cells between G-BM and G-PB (n = 20). Panel **E** shows the ratio of Tcon cells to CD4^+^CD25^high^CD127^-/Low^ Treg cells in G-BM and G-PB (n = 20). *P ≤ 0.05 by Wilcoxon signed-rank test (median with interquartile range).

We found no statistically significant differences comparing G-BM and G-PB with respect to the proportions of CD4+ T cells with a conventional T cell phenotype (CD4^+^CD25^low^CD127^+^; 75.60% ± 5.41% vs. 73.99% ± 7.97%, P = 0.247, Figure 5A) and Th17 cells (3.86% ± 1.96% vs. 2.79% ± 1.40%, p = 0.126, Figure 5B). The proportions of Th1 cells in CD4^+^ T cells were significantly higher in G-BM compared to G-PB (39.51% ± 12.49% vs. 25.17% ± 9.09%, p < 0.001, Figure 5C). However, similar to the differences in regulatory T cells between G-BM and G-PB, the absolute numbers of conventional T cells, Th17 cells and Th1 cells were significantly lower in G-BM than those in G-PB (P < 0.0001, Figure 5D).

In addition to comparisons of individual subsets of T cells between G-BM and G-PB, it is important to examine the relative balance of the effector and regulatory arms of the immune system. The ratio of CD4^+^CD25^low^CD127^+^ conventional T cells to CD4^+^CD25^high^CD127^-/low^ regulatory T cells was significantly lower in G-BM compared to that in G-PB (22.27 ± 20.55 vs. 30.37 ± 14.67, P = 0.014, Figure 5E). In addition, the ratio of CD4^+^CD25^low^CD127^+^ conventional T cells to CD4^+^CD25^high^CD127^-/low^CD62L^+^ regulatory T cells was also lower in G-BM compared to that in G-PB (35.28 ± 29.11 vs. 78.02 ± 57.02, P = 0.008). The ratios of Th17 cells to CD4^+^CD25^high^CD127^-/low^ regulatory T cells were comparable between G-BM and G-PB (P = 0.970).

### Correlations of regulatory T cells between G-BM and G-PB

**Figure 6:**
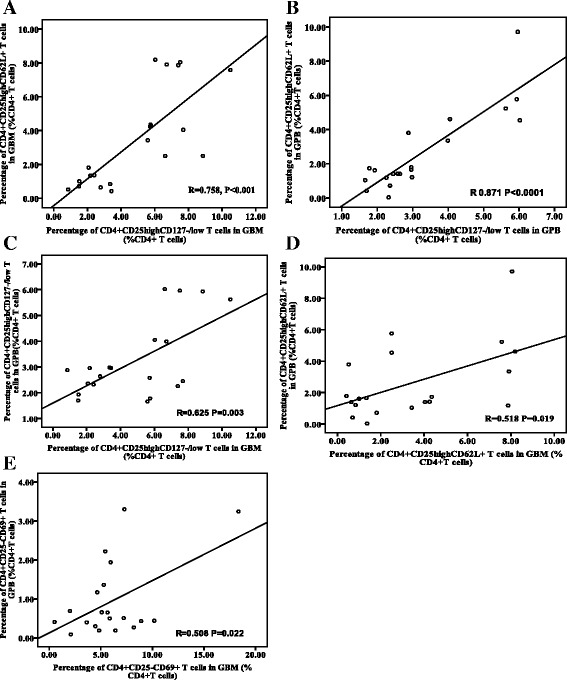
Correlations of CD4^+^CD25^high^CD127^-/low^ and CD4^+^CD25^high^CD62L^+^ regulatory T cells. Panels **A**-**B**. Correlations of CD4^+^CD25^high^CD127^-/low^ and CD4^+^CD25^high^CD62L^+^ regulatory T cells in G-BM **(A)** and G-PB **(B)**. Panels **C-D**. Correlations of CD4^+^CD25^high^CD127^-/low^ **(C)** and CD4^+^CD25^high^CD62L^+^ regulatory T cells **(D)** and CD4^+^CD25^−^CD69^+^ regulatory T cells **(E)** between G-BM and G-PB.

Positive correlations were found between the proportions of CD4^+^CD25^high^CD127^-/low^ regulatory T cells and CD4^+^CD25^high^CD62L^+^ regulatory T cells in G-BM (R = 0.758, P < 0.001, Figure 6A) and in G-PB (R = 0.871, P < 0.001, Figure 6B), but not for CD4^+^CD25^−^CD69^+^ regulatory T cells in G-BM (R = 0.385, P = 0.094) and in G-PB (R = 0.134, P = 0.575).

Finally, positive correlations were found between G-BM and G-PB in the proportions of CD4^+^CD25^high^CD127^-/low^ regulatory T cells (R = 0.625, P = 0.003, Figure 6C), the proportions of CD4^+^CD25^high^CD62L^+^ regulatory T cells (R = 0.518, P = 0.019, Figure 6D), and the proportions of CD4^+^CD25^−^CD69^+^ regulatory T cells (R = 0.508, P = 0.022, Figure 6E).

## Discussion

This is the first study to explore the differences between G-BM and G-PB with regard to the Treg cell contents as well as the balance between effector T cells and regulatory T cells. Our data demonstrated that the proportions of conventional T cells and Th17 cells were comparable; however, the proportions of regulatory T cells and Th1 cells were significantly higher in G-BM compared to G-PB. Therefore, although the absolute number of regulatory T cells, conventional T cells, Th17 cells, or Th1 cells were significantly lower in G-BM compared to G-PB, the ratio of conventional T cells to CD4^+^CD25^high^CD127^-/low^ Treg cells was significantly lower in G-BM compared to G-PB, which could partially contribute to the lower incidence of acute GVHD in G-BM transplantation compared to G-PB transplantation.

The CD4^+^CD25^high^CD127^-/low^ Treg subsets could mediate peripheral T-cell homeostasis and contribute to self-tolerance [25]. Not only the frequencies of CD4^+^CD25^high^CD127^-/low^ Treg cells but also those of CD4^+^CD25^high^CD127^-/low^CD62L^+^ Treg cells were significantly higher in G-BM compared to G-PB. In addition, the fraction of proliferating CD4^+^CD25^high^Foxp3^+^ T cells was significantly higher in G-BM than in G-PB, which might contribute to the higher frequencies of regulatory T cells in G-BM compared to G-PB. Zou WP and colleagues demonstrated that the bone marrow is a reservoir for CD4^+^CD25^+^ regulatory T cells that traffic by means of CXCL12/CXCR4 signaling. G-CSF reduces the expression of human bone marrow CXCL12 in vivo, which is associated with the mobilization of bone marrow Treg cells to the peripheral blood in human volunteers [17]. Boyer O and colleagues found that the frequencies of Treg cells were still significantly lower in G-PB compared to BM transplants. In addition, G-CSF administration and leukapheresis were found to contribute to the loss of the CD62L^+^ Treg cell subset [16]. Consistent with Boyer O et al., our data demonstrated that the expression of CD62L on CD4^+^CD25^high^CD127^-/low^ Treg cells was comparable in G-PB or in G-BM, confirming that G-CSF administration contributes to the loss of CD62L on Treg cells. In addition, the lower levels of CCR7 in CD45RA^+^HLADR^−^ Treg cells in G-BM also indicate a regulatory influence of G-CSF on human Treg-cell migration and suggests a role in the redistribution of Treg cell subsets to the peripheral blood, involving CD62L, CCR7, and CXCL12/CXCR4 signals. It has been suggested that CD62L^+^ Treg cells may have higher immunosuppressive properties in vitro than CD62L^−^ Treg cells [10] and possess the capacity to control GVHD [11,26]. CD69 deficiency leads to diminished levels of TGF-β that contribute to an enhanced immune response, resulting in increased inflammation in a collagen-induced arthritis model [12,27]. Therefore, our data suggested that the higher frequencies of CD4^+^CD25^high^CD127^-/low^CD62L^+^ Treg cells as well as the higher expression of CD69 on Treg cells in G-BM would help maintain the hyporesponsiveness of G-BM compared to G-PB. In addition, distinct human Treg subsets have been described by analyzing cell-surface markers, such as CD45RA and HLADR. Of these, only the CD45RA^+^ naive cells within the CD4 + CD25^high^ T-cell compartment were involved, as only this subpopulation homogeneously expressed CD62L, CCR7, cytotoxic T lymphocyte–associated antigen-4 (CTLA-4), and FOXP3, produced no inflammatory cytokines and maintained robust suppressive activity after expansion [28]. The Treg compartment in patients developing acute GVHD also showed a marked depletion of CD45RA^+^HLADR^−^“naive” Treg cells compared with tolerant patients [24]. Therefore, the higher percentage of “naive” Treg cells in G-BM compared to those in G-PB might indicate higher immunosuppressive properties. However, the results of the in vitro Treg cell suppression assay were comparable between G-BM and G-PB. The limited number of samples studied and the selection of CD4+ CD25+ Tregs using the Regulatory T cell Isolation kit (Miltenyi Biotec) rather than fluorescence-activated cell sorting might affect the cell suppressive activities reported herein. In addition, an imbalance in the effector and regulatory CD4 T cells is associated with GVHD after HSCT using a reduced intensity conditioning regimen and alemtuzumab [29]. The high frequency of CD4^+^CD25^−^CD69^+^ T cells is correlated with a low risk of acute GVHD in allotransplants [8]. Thus, the higher ratio of Tregs to conventional T cells as well as higher frequencies of CD4^+^CD25^−^CD69^+^ T cells in G-BM compared to G-PB would support the lower occurrence of acute GVHD post-G-BM transplantation compared to G-PB transplantation [2].

Correlations were found in CD4^+^CD25^high^CD127^-/low^ Treg cells and CD4^+^CD25^high^CD62L^+^ Treg cells but not in CD4^+^CD25^−^CD69^+^ T cells between G-BM and G-PB, suggesting that the CD4^+^CD25^high^CD127^-/low^ Treg cells and CD4^+^CD25^high^CD62L^+^ Treg cells in G-PB might derive from BM and might overlap each other; however, the development of CD4^+^CD25^−^CD69^+^ T cells in G-PB had no direct relevance to those in G-BM, which will need to be confirmed in the future.

Although acute GVHD has been classically assumed to be Th1-mediated on the basis of findings in animal models, we now face a more complex scenario involving possible roles of newly identified Th17 cells as well as Treg cells in GVHD [30]. The differential expression of chemokine receptors and selectin ligands on each Th subset may ensure the unique ability of each Th subset to induce tissue-specific GVHD [31], and the absence of regulatory T-cell control of Th1 and Th17 cells is responsible for the autoimmune-mediated pathology in chronic graft-versus-host disease [32]. Thus, it appears that a delicate balance among Th1, Th2, Th17, and Treg cells after transplantation is an important determinant of the severity, manifestation, and tissue distribution of GVHD. In this study, the ratio of Th17 cells and Treg cells were comparable between G-BM and G-PB, but the percentages of Th1 in G-BM were higher compared with G-PB. It is not clear whether the Th1 cells could contribute to the prevention or initiation of GVHD post-transplantation [33,34]. Yi et al. have demonstrated that the absence of Th17 cells leads to augmented Th1 differentiation and exacerbated acute GVHD [31]. Waller et al. showed that donor bone marrow precursors of plasmacytoid dendritic cells (pre-pDCs) can activate donor T cells toward Th1 immune polarization, then induce indoleamine 2,3-dioxygenase synthesis to limit GVHD by altering the balance between donor Treg and inflammatory T cells [33]. A better understanding of the dynamic process of reciprocal differentiation of Th and regulatory cell subsets as well as their complex interactions would help establish novel graft engineering that modulates the fine balance between Th and Treg subsets to improve the outcome of allo-HSCT.

## Conclusions

In summary, this study demonstrated that higher frequencies of regulatory T cells and a decreased ratio of effector T cells to regulatory T cells in G-BM would lay part of the immunological groundwork for a lower occurrence of acute or chronic GVHD post-G-BM transplantation compared to G-PB transplantation. Conducting a large cohort study in a prospective manner would be warranted to compare the different clinical outcomes between G-BM and G-PB transplants and their correlation with different Th and Treg subsets within G-BM or G-PB allografts.

## Additional file

Additional file 1: Figure S1. The functional capacities and ontogeny relationships among different regulatory T cells. A common ontogenic pathway might exist between the CD4^+^CD25^high^CD62L^+^ regulatory T cells and CD4^+^CD25^high^CD127^-/low^ regulatory T cells. Both CD4^+^CD25^high^CD62L^+^ regulatory T cells and CD4^+^CD25^high^CD127^-/low^ regulatory T cells can originate from the thymus or mature CD4^+^CD25^high^ T lymphocytes, and there may be overlap between these two regulatory T cells populations. A phenotypic analysis of the CD4^+^CD25^high^CD127^-/low^ regulatory T cell compartment revealed 3 distinct populations (CD45RA^+^HLADR^−^, CD45RA^−^HLADR^+^, CD45RA^−^HLADR^−^). CD4^+^CD25^−^CD69^+^ T cells are generated from mature CD4^+^CD25^−^ T lymphocytes after TGF-β induction. CD69 can be persistently expressed by these cells without CD25 expression. All of these three regulatory T cells populations can inhibit effector T cell function to reduce inflammation and therefore play immunoregulatory roles.
