# Supplementary figures and images for: Higher frequency of regulatory T cells in granulocyte colony-stimulating factor (G-CSF)-primed bone marrow grafts compared with G-CSF-primed peripheral blood grafts
Source: J Transl Med. 2015 May 7;13:145. doi: 10.1186/s12967-015-0507-z (PMC4490623; doi:10.1186/s12967-015-0507-z)

**FigureS1. The functional capacities and ontogeny relation of different regulatory T cells.**

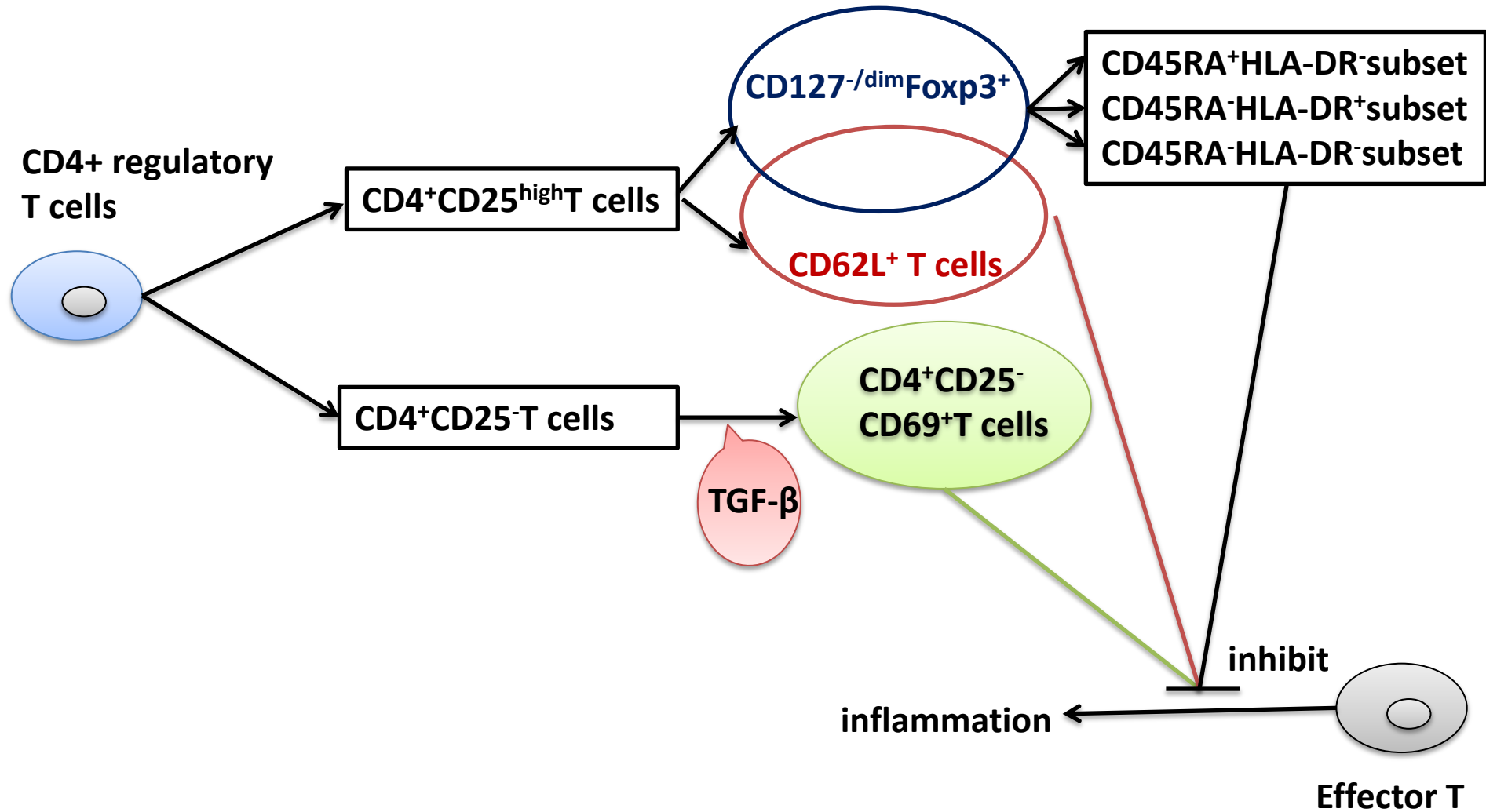

Supplement: Additional file 1: Figure S1. — The functional capacities and ontogeny relationships among different regulatory T cells. A common ontogenic pathway might exist between the CD4+CD25highCD62L+ regulatory T cells and CD4+CD25highCD127-/low regulatory T cells. Both CD4+CD25highCD62L+ regulatory T cells and CD4+CD25highCD127-/low regulatory T cells can originate from the thymus or mature CD4+CD25high T lymphocytes, and there may be overlap between these two regulatory T cells populations. A phenotypic analysis of the CD4+CD25highCD127-/low regulatory T cell compartment revealed 3 distinct populations (CD45RA+HLADR−, CD45RA−HLADR+, CD45RA−HLADR−). CD4+CD25−CD69+ T cells are generated from mature CD4+CD25− T lymphocytes after TGF-β induction. CD69 can be persistently expressed by these cells without CD25 expression. All of these three regulatory T cells populations can inhibit effector T cell function to reduce inflammation and therefore play immunoregulatory roles. [file 12967_2015_507_MOESM1_ESM.pdf]
